# Factors associated with procedural difficulty and major adverse events during transcatheter closure of the patent ductus arteriosus

**DOI:** 10.1371/journal.pone.0354122

**Published:** 2026-07-16

**Authors:** Araya Tipsungnoen, Jirayut Jarutach, Supaporn Roymanee, Kanjarut Wongwaitaweewong, Rujira Buntharikpornpun, Suppalak Puttarak, Kemmapon Chumchuen

**Affiliations:** 1 Division of Pediatric Cardiology, Department of Pediatrics, Faculty of Medicine, Prince of Songkla University, Songkhla, Thailand; 2 Department of Clinical Research and Medical Data Science, Faculty of Medicine, Prince of Songkla University, Songkhla, Thailand; Ataturk University Faculty of Medicine, TÜRKIYE

## Abstract

Transcatheter closure of the patent ductus arteriosus (PDA) is widely accepted as an alternative to surgical ligation. However, major complications are uncommon. Therefore, procedural difficulty, including device repositioning or change, may provide additional insight into technical challenges encountered during PDA closure. We aimed to identify the clinical and anatomical risk factors associated with procedural difficulty or major adverse events during transcatheter PDA closure and to document the prevalence and types of complications encountered in a single tertiary center. This retrospective cohort study included 341 patients who underwent a transcatheter PDA closure between 2010 and 2023. A composite primary outcome, defined as procedural difficulty or major adverse events, included device malposition requiring reposition or change, unsuccessful device deployment, device embolization, hemodynamic instability requiring intervention, or unplanned surgical conversion. Demographic, anatomical, and hemodynamic variables were analyzed. Univariate and multivariate logistic regression analyses were performed to identify independent predictors. Procedural difficulty or major adverse events occurred in 49 patients (14.3%). There was no procedure-related mortality. Device embolization occurred in three patients (0.8%), all requiring surgical ligation. Multivariate analysis revealed that a minimum PDA diameter >5 mm (odds ratio [OR] = 2.75, *P* = 0.013), PDA length >8 mm (OR = 2.81, *P* = 0.009), and non-type A (non-conical) duct morphology (OR = 2.88, *P* = 0.025) were independently associated with the composite outcome. In conclusion, larger PDA diameter, longer ductal length, and non-conical morphology were significantly associated with increased procedural difficulty or major adverse events during transcatheter PDA closure. These findings highlight the importance of detailed anatomical assessment in patients undergoing transcatheter PDA closure.

## Introduction

The ductus arteriosus (DA) is a vital fetal blood vessel connecting the aorta to the pulmonary artery. After birth, the DA typically undergoes active constriction followed by gradual closure. However, incomplete processing results in a condition known as patent ductus arteriosus (PDA).

The incidence of isolated PDAs is approximately 3–8 cases per 10,000 live births in full-term infants [1,2]. In adults, the prevalence decreases to approximately 0.05%, where it usually presents as an isolated defect. Transcatheter PDA closure is currently the preferred treatment modality due to high success rate, low morbidity, and faster recovery compared to surgical ligation. Despite these advantages, the procedure is not without risks, with complication rates ranging from 2–7% [3–9]. Major complications include device embolization and protrusion, which potentially lead to significant obstruction of the left pulmonary artery or aorta, necessitating urgent surgical intervention. Other serious risks include hemolysis requiring blood transfusion due to residual shunting and cardiac arrhythmias. Minor complications include small hematomas and temporary loss of the dorsalis pedis pulse.

To further reduce and manage procedure-related complications, we aimed to identify the clinical and anatomical risk factors associated with adverse outcomes of PDA device closure and document the complications observed at our center.

## Methods

This retrospective cohort study was conducted using the hospital medical records of all patients who underwent interventional PDA device closures between 2010 and 2023. The study was approved by the Institutional Medical Human Research Ethics Committee (REC.67-080-1-1). Records with incomplete data were excluded to maintain data integrity. Primary coil closures were excluded due to their limitations, including use only in small PDAs, whereas occlusive devices overcome these limitations and are therefore preferred. Data were accessed for research purposes between March 2024 and June 2024. All data were anonymized prior to analysis, and the authors did not have access to information that could identify individual participants during or after data collection. As the data were anonymized, individual patient consent for data collection was waived. This study involved a comprehensive examination of patient demographics, clinical profiles, echocardiographic and cardiac catheterization findings, interventional closure methods, and intermediate-term outcomes.

### Cardiac catheterization

Written informed consent was obtained from all patients undergoing transcatheter closure under general anesthesia, with routine heparinization and intravenous antibiotic prophylaxis. Both femoral arterial and venous access were routinely obtained to facilitate comprehensive hemodynamic assessment. An aortogram was obtained from the right anterior oblique and left lateral views to accurately assess the size, shape, and length of the PDA during peak systole. The Krichenko classification system [10] was employed to determine duct morphology and guide the selection of device design and size based on angiographic measurements. The approach technique, determined by the type of device and operator preference, included either an antegrade or a retrograde approach. In the antegrade approach, the duct was crossed from the pulmonary artery and exchanged for a long venous delivery sheath, whereas in the retrograde approach, the duct was accessed from the aorta prior to sheath exchange.

### PDA device closure

For most ductal morphologies, a conventional duct occluder sized approximately 2 mm larger than the minimum PDA diameter was selected. In tubular Type C ducts, an Amplatzer Vascular Plug II (Abbott Medical, Plymouth, MN, USA), sized at least 1.5–2 times the duct diameter, was preferentially used. Device position and stability were confirmed by repeat angiography prior to release, ensuring absence of significant protrusion into the aorta or left pulmonary artery. If instability or malposition was observed, the device was retrieved and replaced as necessary. Following device release, chest radiography was performed to confirm device position. Echocardiographic follow-up was conducted at 24 h postprocedure and subsequently at 1, 6, and 12 months.

### Definitions

Because major complications after transcatheter PDA closure were infrequent, a composite primary outcome was used to allow meaningful multivariable analysis. The composite outcome consisted of procedural difficulty or major adverse events, defined as device malposition requiring reposition or change, unsuccessful device deployment (procedure aborted), device embolization, hemodynamic instability requiring intervention, and unplanned surgical conversion. Hemodynamic instability was defined as any intra-procedural event requiring urgent intervention, including hypotension requiring inotropic support, significant arrhythmia requiring treatment, or oxygen desaturation requiring escalation of respiratory support. Procedural success was defined as successful device implantation without any component of the composite outcome. Major adverse events were reported descriptively and included device embolization, surgical conversion, significant vascular obstruction, or death.

### Statistical analysis

Continuous variables are presented as mean ± standard deviation or median with interquartile range, depending on distribution. Categorical variables are reported as frequencies and percentages. Comparisons between groups were performed using Student’s t-test or Mann–Whitney U test for continuous variables and chi-square or Fisher’s exact test for categorical variables, as appropriate. Variables with P < 0.10 on univariate analysis, together with clinically relevant covariates, were entered into a multivariable logistic regression model to identify independent predictors of the composite outcome. PDA diameter and PDA length were analyzed as categorical variables using clinically relevant thresholds (>5 mm and >8 mm, respectively) [11]. Odds ratios (ORs) with 95% confidence intervals (CIs) were reported. All tests were two-sided, and P < 0.05 was considered statistically significant. Statistical analyses were performed using R software version 4.2.3. (R Foundation for Statistical Computing, Vienna, Austria).

## Results

A total of 360 patients underwent transcatheter PDA device closure during the study period. Nineteen patients were excluded (nine due to incomplete data and ten due to coil closure), leaving 341 patients for final analysis.

The median age was 3.8 years, with a male-to-female ratio of 1:2.6. Chromosomal abnormalities were present in 30 patients (8.8%), with Down syndrome being the most common. PDA was an isolated lesion in 91% of cases.

Device closure was successful in 331 patients (97.1%). Device deployment was unsuccessful in seven patients (2.1%), and device embolization occurred in three patients (0.8%), all of whom required surgical device retrieval and PDA ligation. The composite outcome of procedural difficulty or major adverse events occurred in 49 patients (14.3%) and is summarized in Table 1. No procedure-related mortality was observed. The most frequent adverse events were device malposition requiring reposition or device change (6.7%), hemodynamic instability requiring intervention (2.9%), and unsuccessful device deployment (2.1%). Among the 23 patients with device malposition, device upsizing was required in all cases and device type was changed in 11 patients.

**Table 1:** Components of procedural difficulty and adverse events during transcatheter PDA closure (n = 49).

Event	n (%)
Device malposition	23 (46.9)
Hemodynamic instability	10 (20.4)
Unsuccessful device deployment	7 (14.3)
LPA stenosis	5 (10.2)
Vascular complication	4 (8.2)
Embolization	3 (6.1)
Acquired CoA	2 (4.1)
Residual shunt	2 (4.1)
Valvular regurgitation	1 (2.0)

Values are presented as number (%). Some patients experienced more than one event; therefore, percentages may exceed 100%.

Cases of left pulmonary artery (LPA) stenosis and acquired coarctation of aorta (CoA) were initially managed with echocardiographic follow-up. In patients with persistent or significant obstruction, balloon angioplasty was considered. Hemodynamic instability was managed with standard supportive measures, including inotropic support, or temporary procedural interruption, as appropriate.

On univariate analysis, patients with the composite outcome had significantly shorter height. Anatomically, these patients were more likely to have non-type A PDA morphology, larger minimum PDA diameter, larger pulmonary artery end diameter, and longer ductal length. Hemodynamic assessment revealed higher pulmonary artery pressure, wider pulse pressure, and increased pulmonary-to-systemic flow ratio (Qp/Qs) in the composite outcome group (Table 2).

**Table 2:** Comparison of baseline characteristics and procedural variables according to adverse outcome status.

Variable	All (n = 341)	No Adverse Outcome (n = 292)	Adverse Outcome (n = 49)	*P* value
Age, years	3.8 (1.7–13.1)	3.9 (1.8–12.6)	2.4 (1.3–13.8)	0.117
Female, n (%)	248 (72.7)	213 (72.9)	35 (71.4)	0.962
Height, m	1.0 (0.8–1.4)	1.0 (0.8–1.4)	0.8 (0.7–1.2)	0.030*
Weight, kg	14.0 (9.5–36.7)	14.1 (9.7–36.0)	12.5 (7.7–40.0)	0.097
BSA, m^2^	0.6 (0.5–1.2)	0.6 (0.5–1.2)	0.6 (0.4–1.2)	0.077
PDA ampulla diameter, mm	9.0 (7.0–12.0)	9.0 (7.0–12.0)	9.3 (8.0–13.2)	0.124
PDA minimum diameter, mm	3.8 (2.5–5.0)	3.6 (2.5–5.0)	4.7 (3.6–7.4)	< 0.001*
PDA pulmonary end, mm	3.9 (2.7–5.0)	3.6 (2.5–5.5)	4.7 (3.9–7.4)	< 0.001*
PDA length, mm	6.0 (5.0–8.0)	6.0 (5.0–7.7)	8.0 (6.0–10.0)	< 0.001*
Type A PDA, n (%)	304 (90.2)	266 (92.0)	38 (79.2)	0.014*
Systolic PAP, mmHg	30.0 (25.0–41.0)	29.0 (24.0–39.0)	37.0 (26.0–52.0)	0.003*
Diastolic PAP, mmHg	16.0 (13.0–22.0)	16.0 (13.0–21.0)	18.0 (14.0–23.0)	0.039*
Mean PAP, mmHg	22.5 (19.0–30.0)	22.0 (19.0–29.0)	27.0 (21.0–32.0)	0.001*
Pulse pressure, mmHg	44.0 (37.0–52.0)	43.0 (37.0–51.0)	46.0 (40.0–55.0)	0.026*
Qp/Qs	1.7 (1.4–2.4)	1.6 (1.4–2.2)	1.8 (1.6–3.4)	0.007*
Rp/Rs	0.1 (0.1–0.2)	0.1 (0.1–0.2)	0.2 (0.1–0.2)	0.165
PVRi (WU.m^2^)	1.3 (0.8–2.3)	1.3 (0.8–2.2)	1.7 (1.0–2.6)	0.092
Genetic disease, n (%)	30 (8.8)	24 (8.2)	6 (12.2)	0.410

Data are presented as median (interquartile range) and number (%) for categorical variables.

Adverse outcome was defined as a composite of procedural difficulty or major adverse events.

BSA body surface area; PAP pulmonary artery pressure; PDA patent ductus arteriosus.

*P value < 0.05.

In the multivariate logistic regression analysis (Table 3), the independent variables comprised the minimum PDA diameter, PA end diameter, PDA length, PDA morphology, age, BSA, and presence of genetic disease; while the dependent variables were the outcomes of the PDA procedures. A minimum PDA diameter greater than 5 mm, with an odds ratio (OR) of 2.75 (*P* value = 0.013), a PDA length greater than 8 mm, with an OR 2.81 (*P* value = 0.009), non-Type A PDA morphology with an OR of 2.88 (*P* value = 0.025) were independently associated with the composite outcome.

**Table 3:** Multivariable logistic regression analysis of factors associated with adverse outcomes during PDA device closure.

Variables	Odds ratio (95% CI)	*P* value
PDA minimum diameter >5 mm.	2.75 (1.24–6.12)	0.013*
PDA length >8 mm	2.81 (1.32–6.00)	0.009*
Non-type A PDA	2.88 (1.14–7.26)	0.025*
BSA	0.48 (0.19–1.19)	0.099
Genetic disease	1.84 (0.62–5.47)	0.288

BSA body surface area; PDA patent ductus arteriosus.

*P value < 0.05.

## Discussion

This large single-center study evaluated clinical and anatomical predictors of procedural difficulty and major adverse events during transcatheter PDA closure. Consistent with prior reports, overall procedural success was high, with successful device implantation achieved in 97.1% of cases, within the reported range of 92–98.6% [3–9].

The observed composite outcome rate of 14.3% appears higher than that reported in previous studies [3–9,12,13]. However, this difference likely reflects our deliberate inclusion of procedural difficulty, such as device repositioning or change, together with major adverse events, rather than reporting only traditional complications. While device repositioning is often considered part of routine procedural optimization, it represents a clinically relevant marker of technical complexity and resource utilization. Inclusion of these events provides a more comprehensive depiction of real-world practice, particularly in anatomically challenging PDAs. This approach may be especially informative for early-career interventionists, offering practical guidance for patient selection and procedural planning.

Device embolization remains a rare but serious complication. In our cohort, the embolization rate was 0.8% (3 of 341 patients), comparable to previously reported rates ranging from 0.3% to 1.1% [3,4,14]. These findings reaffirm the overall safety and reliability of transcatheter PDA closure across diverse practice settings.

An important finding of this study is the identification of specific anatomical predictors associated with procedural difficulty or major adverse events. A minimum PDA diameter greater than 5 mm independently increased the risk nearly threefold (OR 2.75, *P* = 0.013). Larger ducts typically require larger devices, which may be more difficult to stabilize and more prone to malposition or embolization. Although no standardized cutoff values have been previously established, Mumtaz et al. similarly demonstrated that increasing PDA diameter was significantly associated with device malposition and embolization [15]. Our results suggest that a minimum diameter of 5 mm may represent a clinically meaningful threshold warranting heightened procedural caution.

We also identified PDA length greater than 8 mm as an independent predictor (OR 2.81, *P* = 0.009). This finding has practical implications, as conventional duct occluders are generally limited in length, and longer PDAs often necessitate alternative devices such as vascular plugs or extra-large occluders. These devices may require greater technical expertise and carry increased risk of protrusion into adjacent vessels, contributing to procedural complexity.

Furthermore, non-type A (non-conical) duct morphology was significantly associated with adverse outcomes (OR 2.88, *P* = 0.025). Non-conical PDAs, including tubular and irregular morphologies, provide less predictable anchoring for standard devices and have been previously linked to higher rates of device protrusion and instability [15,16]. Our findings reinforce the importance of detailed angiographic assessment of duct morphology to guide device selection and procedural planning.

In addition to anatomical factors, hemodynamic parameters such as pulmonary artery pressure and Qp/Qs were significantly higher in patients with adverse outcomes on univariate analysis. These findings likely reflect increased shunt volume and overall hemodynamic burden, particularly in larger or more complex PDAs. Although these variables were not independent predictors in multivariable analysis, they may still contribute to procedural complexity and technical challenges during device closure.

Although most cases in our cohort involved isolated PDA, the presence of associated cardiac conditions may further influence pulmonary hemodynamics and procedural difficulty. These observations highlight the importance of comprehensive hemodynamic assessment in addition to anatomical evaluation during preprocedural planning.

Taken together, these findings suggest that procedural difficulty and adverse outcomes are primarily associated with anatomical complexity, while hemodynamic factors may also play a contributory role in selected cases. Comprehensive preprocedural assessment of both anatomical and hemodynamic characteristics is therefore essential to guide device strategy and improve procedural safety, particularly in technically challenging PDAs.

### Limitations

This study has several limitations. First, its retrospective, single-center design may limit generalizability. Second, the wide age range of the study population may introduce heterogeneity, as procedural considerations and outcomes can differ between pediatric and adult patients; therefore, the findings may not be fully representative of either group individually. Third, PDA measurements were based primarily on angiographic assessment without systematic correlation with echocardiographic parameters, which may have introduced measurement variability. In addition, procedural techniques and device availability evolved over the study period, potentially influencing outcomes. Finally, inclusion of procedural difficulty in the composite outcome, while intended to reflect real-world technical complexity, may limit direct comparison with studies reporting only major complications.

## Conclusions

This study identifies key anatomical factors associated with procedural difficulty and major adverse events during transcatheter PDA closure. A minimum PDA diameter >5 mm, PDA length >8 mm, and non-type A duct morphology were independently associated with increased risk. These findings highlight the importance of detailed anatomical assessment in patients undergoing transcatheter PDA closure.
