# Supplementary material for: Factors associated with procedural difficulty and major adverse events during transcatheter closure of the patent ductus arteriosus
Source: PLoS One. 2026 Jul 16;21(7):e0354122. doi: 10.1371/journal.pone.0354122 (PMC13374907; doi:10.1371/journal.pone.0354122)
Supplement: Attachment — Submitted filename: Response to Reviewers.docx [file pone.0354122.s002.docx]

**Manuscript ID:** PONE-D-26-07135
**Title:** Factors associated with procedural difficulty and major adverse events during transcatheter closure of the patent ductus arteriosus

**Response to the Editor and Reviewers**

We sincerely thank the Academic Editor and both reviewers for their thoughtful comments and constructive suggestions. We have carefully revised the manuscript to improve clarity, methodological transparency, and alignment between results and conclusions. All changes have been incorporated into the revised manuscript and highlighted accordingly.

Below, we provide a point-by-point response to each comment.

**Reviewer #1**

**Comment 1:**

*Introduction: PDA device closure is the preferred method of PDA treatment.*

**Response:**
Thank you for this helpful suggestion. We have revised the Introduction to clearly state that transcatheter PDA closure is currently the preferred treatment modality due to its high success rate, lower morbidity, and faster recovery compared with surgical ligation.

**Comment 2:**

*How were the embolisations, LPA and CoA stenosis managed?*

**Response:**
We appreciate this important comment. We have clarified management strategies in the **Results section**:

- Device embolization cases were managed with **surgical device retrieval and PDA ligation**.
- Cases of **left pulmonary artery (LPA) stenosis** and **acquired coarctation of the aorta (CoA)** were initially managed with **echocardiographic follow-up**, and **balloon angioplasty was considered** in patients with persistent or significant obstruction.

**Comment 3:**

*What was the definition of hemodynamic instability? How was it managed?*

**Response:**
Thank you for raising this point. We have addressed this by:

- Adding a clear **definition in the Definitions section**:

“Hemodynamic instability was defined as any intra-procedural event requiring urgent intervention, including hypotension requiring inotropic support, significant arrhythmia requiring treatment, or oxygen desaturation requiring escalation of respiratory support.”

- Describing management in the **Results section**, including supportive measures such as:
  - inotropic support
  - temporary procedural interruption

**Comment 4:**

*There are too many conclusions, please focus only on those that are proved by the results.*

**Response:**
We appreciate this important suggestion. We have revised both the **Abstract and Conclusions sections** to ensure that all statements are directly supported by the results.

Specifically:

- Conclusions now focus only on **independent predictors identified in multivariable analysis** (PDA diameter >5 mm, PDA length >8 mm, and non-type A morphology)
- Interpretative or general statements not directly supported by the data (e.g., device strategy or broader clinical implications) have been removed

These revisions ensure a clear alignment between the results and the conclusions.

**Reviewer #2**

**Comment 1:**

*PDA size and body surface area*

**Response:**
Thank you for this comment. Both **PDA size** and **body surface area (BSA)** were included in the analysis:

- PDA diameter was identified as an **independent predictor** in multivariable analysis
- BSA was included in the regression model but was **not independently associated** with the composite outcome

This has been clarified in the **Results section**.

**Comment 2:**

*Types of PDA and technical difficulties*

**Response:**
We agree with this important point. We have addressed it by:

- Including **PDA morphology (Krichenko classification)** in the analysis
- Demonstrating that **non-type A (non-conical) morphology** was independently associated with procedural difficulty

This is further discussed in the **Discussion section**, emphasizing its relevance to procedural complexity.

**Comment 3:**

*Large PDA and their complication rate*

**Response:**
Thank you. This has been addressed by:

- Identifying **PDA diameter >5 mm** as an independent predictor of the composite outcome
- Expanding the Discussion to explain the relationship between larger duct size and increased procedural complexity

**Comment 4:**

*PDA associated with other congenital heart diseases and its impact on pulmonary artery pressure*

**Response:**
We appreciate this insightful comment. While most patients in our cohort had isolated PDA, we have:

- Noted in the **Discussion section** that associated cardiac conditions may influence pulmonary hemodynamics
- Reported that **pulmonary artery pressure and Qp/Qs were higher in patients with adverse outcomes on univariate analysis**

These findings are presented as **supportive observations**, as they were not independent predictors in multivariable analysis.

**Additional Revisions**

- Revised the **Abstract, Discussion, and Conclusions** to ensure consistency and avoid overinterpretation
- Improved clarity and readability throughout the manuscript
- Ensured alignment between reported results and final conclusions
- Addressed all formatting and reporting requirements according to PLOS ONE guidelines

**Conclusion**

We believe that these revisions have strengthened the manuscript and addressed all reviewer comments. We sincerely thank the reviewers and the editor for their valuable feedback.
