# Supplementary material for: Factors associated with procedural difficulty and major adverse events during transcatheter closure of the patent ductus arteriosus
Source: PLoS One. 2026 Jul 16;21(7):e0354122. doi: 10.1371/journal.pone.0354122 (PMC13374907; doi:10.1371/journal.pone.0354122)
Supplement: Attachment — Submitted filename: Response to Reviewers 2nd.docx [file pone.0354122.s003.docx]

**Manuscript ID:** PONE-D-26-07135
**Title:** Factors associated with procedural difficulty and major adverse events during transcatheter closure of the patent ductus arteriosus

**Response to the Editor and Reviewers**

Reviewer comment:
“What were the results of balloon angioplasties of iatrogenic CoA and LPA stenosis? Was it effective?”

Response:
Thank you for this valuable comment. Among the five patients with left pulmonary artery (LPA) stenosis, spontaneous improvement was observed in three patients during follow-up and no intervention was required. The remaining two patients had peak pressure gradients of approximately 40 mmHg and underwent balloon angioplasty, resulting in hemodynamic improvement with residual gradients of approximately 30 mmHg. These patients remain under follow-up, and further intervention such as LPA stenting may be considered if progressive stenosis or right ventricular hypertension develops.

Regarding iatrogenic coarctation of the aorta, both patients had mild mean pressure gradients of approximately 10–20 mmHg and are currently being monitored clinically for systemic hypertension and progression of the lesion without additional intervention.

As these events were limited in number and detailed long-term follow-up outcomes were beyond the predefined objectives of the present study, we have not incorporated these additional data into the revised manuscript.
