# Supplementary material for: Factors associated with procedural difficulty and major adverse events during transcatheter closure of the patent ductus arteriosus
Source: PLoS One. 2026 Jul 16;21(7):e0354122. doi: 10.1371/journal.pone.0354122 (PMC13374907; doi:10.1371/journal.pone.0354122)
Supplement: Peer review history

# Peer review: Decision Letter 0

**DOI:** 10.1371/journal.pone.0354122.r001

**Authors:** Jennifer Tucker

7 Apr 2026

PONE-D-26-07135

Factors associated with procedural difficulty and major adverse events during transcatheter closure of the patent ductus arteriosus

PLOS One

Dear Dr. jarutach,

Thank you for submitting your manuscript to PLOS ONE. After careful consideration, we feel that it has merit but does not fully meet PLOS ONE’s publication criteria as it currently stands. Therefore, we invite you to submit a revised version of the manuscript that addresses the points raised during the review process.

Please submit your revised manuscript by May 22 2026 11:59PM. If you will need more time than this to complete your revisions, please reply to this message or contact the journal office at plosone@plos.org. When you're ready to submit your revision, log on to https://www.editorialmanager.com/pone/ and select the 'Submissions Needing Revision' folder to locate your manuscript file.

Please include the following items when submitting your revised manuscript:

- A letter that responds to each point raised by the academic editor and reviewer(s). You should upload this letter as a separate file labeled 'Response to Reviewers'.
- A marked-up copy of your manuscript that highlights changes made to the original version. You should upload this as a separate file labeled 'Revised Manuscript with Track Changes'.
- An unmarked version of your revised paper without tracked changes. You should upload this as a separate file labeled 'Manuscript'.

If you would like to make changes to your financial disclosure, please include your updated statement in your cover letter. Guidelines for resubmitting your figure files are available below the reviewer comments at the end of this letter.

If applicable, we recommend that you deposit your laboratory protocols in protocols.io to enhance the reproducibility of your results. Protocols.io assigns your protocol its own identifier (DOI) so that it can be cited independently in the future. For instructions see: https://journals.plos.org/plosone/s/submission-guidelines#loc-laboratory-protocols. Additionally, PLOS ONE offers an option for publishing peer-reviewed Lab Protocol articles, which describe protocols hosted on protocols.io. Read more information on sharing protocols at https://plos.org/protocols?utm_medium=editorial-email&utm_source=authorletters&utm_campaign=protocols.

As the corresponding author, your ORCID iD is verified in the submission system and will appear in the published article. PLOS supports the use of ORCID, and we encourage all coauthors to register for an ORCID iD and use it as well. Please encourage your coauthors to verify their ORCID iD within the submission system before final acceptance, as unverified ORCID iDs will not appear in the published article. *Only*  the individual author can complete the verification step; PLOS staff *cannot*  verify ORCID iDs on behalf of authors.

We look forward to receiving your revised manuscript.

Kind regards,

Jennifer Tucker, PhD

Staff Editor

PLOS One

Journal Requirements:

When submitting your revision, we need you to address these additional requirements.

1.Please ensure that your manuscript meets PLOS ONE's style requirements, including those for file naming. The PLOS ONE style templates can be found at

https://journals.plos.org/plosone/s/file?id=wjVg/PLOSOne_formatting_sample_main_body.pdf and

https://journals.plos.org/plosone/s/file?id=ba62/PLOSOne_formatting_sample_title_authors_affiliations.pdf

2. For studies involving third-party data, we encourage authors to share any data specific to their analyses that they can legally distribute. PLOS recognizes, however, that authors may be using third-party data they do not have the rights to share. When third-party data cannot be publicly shared, authors must provide all information necessary for interested researchers to apply to gain access to the data. (https://journals.plos.org/plosone/s/data-availability#loc-acceptable-data-access-restrictions)

For any third-party data that the authors cannot legally distribute, they should include the following information in their Data Availability Statement upon submission:

1) A description of the data set and the third-party source

2) If applicable, verification of permission to use the data set

3) Confirmation of whether the authors received any special privileges in accessing the data that other researchers would not have

4) All necessary contact information others would need to apply to gain access to the data

3. Please include your tables as part of your main manuscript and remove the individual files. Please note that supplementary tables (should remain/ be uploaded) as separate "supporting information" files.

4.If the reviewer comments include a recommendation to cite specific previously published works, please review and evaluate these publications to determine whether they are relevant and should be cited. There is no requirement to cite these works unless the editor has indicated otherwise.

5. Please review your reference list to ensure that it is complete and correct. If you have cited papers that have been retracted, please include the rationale for doing so in the manuscript text, or remove these references and replace them with relevant current references. Any changes to the reference list should be mentioned in the rebuttal letter that accompanies your revised manuscript. If you need to cite a retracted article, indicate the article’s retracted status in the References list and also include a citation and full reference for the retraction notice.

Additional Editor Comments:

-->The manuscript has been evaluated by two reviewers, and their comments are available below.

The reviewers have requested minor revisions to improve the clarity of reporting and understanding of the study design, including definition of certain terms or standards of care.

Could you please carefully revise the manuscript to address all comments raised?

[Note: HTML markup is below. Please do not edit.]

Reviewers' comments:

Reviewer's Responses to Questions

**Comments to the Author**

1. Is the manuscript technically sound, and do the data support the conclusions?

The manuscript must describe a technically sound piece of scientific research with data that supports the conclusions. Experiments must have been conducted rigorously, with appropriate controls, replication, and sample sizes. The conclusions must be drawn appropriately based on the data presented.

Reviewer #1: Yes

Reviewer #2: Yes

**********

2. Has the statistical analysis been performed appropriately and rigorously?

Reviewer #1: Yes

Reviewer #2: Yes

**********

3. Have the authors made all data underlying the findings in their manuscript fully available?

The PLOS Data policy requires authors to make all data underlying the findings described in their manuscript fully available without restriction, with rare exception (please refer to the Data Availability Statement in the manuscript PDF file). The data should be provided as part of the manuscript or its supporting information, or deposited to a public repository. For example, in addition to summary statistics, the data points behind means, medians and variance measures should be available. If there are restrictions on publicly sharing data—e.g. participant privacy or use of data from a third party—those must be specified.

Reviewer #1: Yes

Reviewer #2: Yes

**********

4. Is the manuscript presented in an intelligible fashion and written in standard English?

PLOS ONE does not copyedit accepted manuscripts, so the language in submitted articles must be clear, correct, and unambiguous. Any typographical or grammatical errors should be corrected at revision, so please note any specific errors here.

Reviewer #1: Yes

Reviewer #2: Yes

**********

5. Review Comments to the Author

Please use the space provided to explain your answers to the questions above. You may also include additional comments for the author, including concerns about dual publication, research ethics, or publication ethics. (Please upload your review as an attachment if it exceeds 20,000 characters)

Reviewer #1: Dear Authors,

The manuscript is quite interesting. Total number of patients is not enough to make a very strong conclusions. Nevertheless, I suggest it is worth publishing, and the paper needs only some minor revision.

A few points:

1. Introduction: PDA device closure is the preferred method of PDA treatment;

2. How were the embolisations, LPA and CoA stenosis managed?;

3. What was the definition of hemodynamic instability? How was it managed?

4. There are too many conclusions, please, focus only on that that are proved by the results.

Reviewer #2: PDA size and body surface area

Types of PDA and technical difficulties

Large PDA and their complications rate

PDA associated with other congenital heart diseases and it's impact on pulmonary artery pressure

**********

6. PLOS authors have the option to publish the peer review history of their article (what does this mean?). If published, this will include your full peer review and any attached files.

If you choose “no”, your identity will remain anonymous but your review may still be made public.

**Do you want your identity to be public for this peer review?**  For information about this choice, including consent withdrawal, please see our Privacy Policy.

Reviewer #1: **Yes:**  Michał Gałeczka

Reviewer #2: No

**********

[NOTE: If reviewer comments were submitted as an attachment file, they will be attached to this email and accessible via the submission site. Please log into your account, locate the manuscript record, and check for the action link "View Attachments". If this link does not appear, there are no attachment files.]

To ensure your figures meet our technical requirements, please review our figure guidelines: https://journals.plos.org/plosone/s/figures

You may also use PLOS’s free figure tool, NAAS, to help you prepare publication quality figures: https://journals.plos.org/plosone/s/figures#loc-tools-for-figure-preparation.

NAAS will assess whether your figures meet our technical requirements by comparing each figure against our figure specifications.

# Author comment: Author response to Decision Letter 1

**DOI:** 10.1371/journal.pone.0354122.r002

9 Apr 2026

Manuscript ID: PONE-D-26-07135

Title: Factors associated with procedural difficulty and major adverse events during transcatheter closure of the patent ductus arteriosus

Response to the Editor and Reviewers

We sincerely thank the Academic Editor and both reviewers for their thoughtful comments and constructive suggestions. We have carefully revised the manuscript to improve clarity, methodological transparency, and alignment between results and conclusions. All changes have been incorporated into the revised manuscript and highlighted accordingly.

Below, we provide a point-by-point response to each comment.

Reviewer #1

Comment 1:

Introduction: PDA device closure is the preferred method of PDA treatment.

Response:

Thank you for this helpful suggestion. We have revised the Introduction to clearly state that transcatheter PDA closure is currently the preferred treatment modality due to its high success rate, lower morbidity, and faster recovery compared with surgical ligation.

Comment 2:

How were the embolisations, LPA and CoA stenosis managed?

Response:

We appreciate this important comment. We have clarified management strategies in the Results section:

• Device embolization cases were managed with surgical device retrieval and PDA ligation.

• Cases of left pulmonary artery (LPA) stenosis and acquired coarctation of the aorta (CoA) were initially managed with echocardiographic follow-up, and balloon angioplasty was considered in patients with persistent or significant obstruction.

Comment 3:

What was the definition of hemodynamic instability? How was it managed?

Response:

Thank you for raising this point. We have addressed this by:

• Adding a clear definition in the Definitions section:

“Hemodynamic instability was defined as any intra-procedural event requiring urgent intervention, including hypotension requiring inotropic support, significant arrhythmia requiring treatment, or oxygen desaturation requiring escalation of respiratory support.”

• Describing management in the Results section, including supportive measures such as:

o inotropic support

o temporary procedural interruption

Comment 4:

There are too many conclusions, please focus only on those that are proved by the results.

Response:

We appreciate this important suggestion. We have revised both the Abstract and Conclusions sections to ensure that all statements are directly supported by the results.

Specifically:

• Conclusions now focus only on independent predictors identified in multivariable analysis (PDA diameter >5 mm, PDA length >8 mm, and non-type A morphology)

• Interpretative or general statements not directly supported by the data (e.g., device strategy or broader clinical implications) have been removed

These revisions ensure a clear alignment between the results and the conclusions.

Reviewer #2

Comment 1:

PDA size and body surface area

Response:

Thank you for this comment. Both PDA size and body surface area (BSA) were included in the analysis:

• PDA diameter was identified as an independent predictor in multivariable analysis

• BSA was included in the regression model but was not independently associated with the composite outcome

This has been clarified in the Results section.

Comment 2:

Types of PDA and technical difficulties

Response:

We agree with this important point. We have addressed it by:

• Including PDA morphology (Krichenko classification) in the analysis

• Demonstrating that non-type A (non-conical) morphology was independently associated with procedural difficulty

This is further discussed in the Discussion section, emphasizing its relevance to procedural complexity.

Comment 3:

Large PDA and their complication rate

Response:

Thank you. This has been addressed by:

• Identifying PDA diameter >5 mm as an independent predictor of the composite outcome

• Expanding the Discussion to explain the relationship between larger duct size and increased procedural complexity

Comment 4:

PDA associated with other congenital heart diseases and its impact on pulmonary artery pressure

Response:

We appreciate this insightful comment. While most patients in our cohort had isolated PDA, we have:

• Noted in the Discussion section that associated cardiac conditions may influence pulmonary hemodynamics

• Reported that pulmonary artery pressure and Qp/Qs were higher in patients with adverse outcomes on univariate analysis

These findings are presented as supportive observations, as they were not independent predictors in multivariable analysis.

Additional Revisions

• Revised the Abstract, Discussion, and Conclusions to ensure consistency and avoid overinterpretation

• Improved clarity and readability throughout the manuscript

• Ensured alignment between reported results and final conclusions

• Addressed all formatting and reporting requirements according to PLOS ONE guidelines

Conclusion

We believe that these revisions have strengthened the manuscript and addressed all reviewer comments. We sincerely thank the reviewers and the editor for their valuable feedback.

Attachment Submitted filename: Response to Reviewers.docx

# Peer review: Decision Letter 1

**DOI:** 10.1371/journal.pone.0354122.r003

**Authors:** Jennifer Tucker, Eyüp Serhat Çalık

18 May 2026

PONE-D-26-07135R1

Factors associated with procedural difficulty and major adverse events during transcatheter closure of the patent ductus arteriosus

PLOS One

Dear Dr. Jarutach,

Thank you for submitting your manuscript to PLOS ONE. After careful consideration, we feel that it has merit but does not fully meet PLOS ONE’s publication criteria as it currently stands. Therefore, we invite you to submit a revised version of the manuscript that addresses the points raised during the review process.

Please submit your revised manuscript by Jul 02 2026 11:59PM. If you will need more time than this to complete your revisions, please reply to this message or contact the journal office at plosone@plos.org. When you're ready to submit your revision, log on to https://www.editorialmanager.com/pone/ and select the 'Submissions Needing Revision' folder to locate your manuscript file.

Please include the following items when submitting your revised manuscript:

- A letter that responds to each point raised by the academic editor and reviewer(s). You should upload this letter as a separate file labeled 'Response to Reviewers'.
- A marked-up copy of your manuscript that highlights changes made to the original version. You should upload this as a separate file labeled 'Revised Manuscript with Track Changes'.
- An unmarked version of your revised paper without tracked changes. You should upload this as a separate file labeled 'Manuscript'.

If you would like to make changes to your financial disclosure, please include your updated statement in your cover letter. Guidelines for resubmitting your figure files are available below the reviewer comments at the end of this letter.

If applicable, we recommend that you deposit your laboratory protocols in protocols.io to enhance the reproducibility of your results. Protocols.io assigns your protocol its own identifier (DOI) so that it can be cited independently in the future. For instructions see: https://journals.plos.org/plosone/s/submission-guidelines#loc-laboratory-protocols. Additionally, PLOS ONE offers an option for publishing peer-reviewed Lab Protocol articles, which describe protocols hosted on protocols.io. Read more information on sharing protocols at https://plos.org/protocols?utm_medium=editorial-email&utm_source=authorletters&utm_campaign=protocols.

As the corresponding author, your ORCID iD is verified in the submission system and will appear in the published article. PLOS supports the use of ORCID, and we encourage all coauthors to register for an ORCID iD and use it as well. Please encourage your coauthors to verify their ORCID iD within the submission system before final acceptance, as unverified ORCID iDs will not appear in the published article. *Only*  the individual author can complete the verification step; PLOS staff *cannot*  verify ORCID iDs on behalf of authors.

We look forward to receiving your revised manuscript.

Kind regards,

Eyüp Serhat Çalık

Academic Editor

PLOS One

Journal Requirements:

If the reviewer comments include a recommendation to cite specific previously published works, please review and evaluate these publications to determine whether they are relevant and should be cited. There is no requirement to cite these works unless the editor has indicated otherwise.

Please review your reference list to ensure that it is complete and correct. If you have cited papers that have been retracted, please include the rationale for doing so in the manuscript text, or remove these references and replace them with relevant current references. Any changes to the reference list should be mentioned in the rebuttal letter that accompanies your revised manuscript. If you need to cite a retracted article, indicate the article’s retracted status in the References list and also include a citation and full reference for the retraction notice.

Additional Editor Comments (if provided):

I would like to congratulate the authors on their valuable work. I would also like to thank them for the appropriate revisions made in the first revision draft and for their concise responses to the comments. The manuscript has been re-evaluated by the previous reviewers, and minor additional revisions have been suggested. We eagerly await the submission of your manuscript after you have made the appropriate revisions and provided point-by-point responses to the comments. Best of luck.

[Note: HTML markup is below. Please do not edit.]

Reviewers' comments:

Reviewer's Responses to Questions

**Comments to the Author**

1. If the authors have adequately addressed your comments raised in a previous round of review and you feel that this manuscript is now acceptable for publication, you may indicate that here to bypass the “Comments to the Author” section, enter your conflict of interest statement in the “Confidential to Editor” section, and submit your "Accept" recommendation.

Reviewer #1: (No Response)

Reviewer #2: All comments have been addressed

**********

2. Is the manuscript technically sound, and do the data support the conclusions?

The manuscript must describe a technically sound piece of scientific research with data that supports the conclusions. Experiments must have been conducted rigorously, with appropriate controls, replication, and sample sizes. The conclusions must be drawn appropriately based on the data presented.

Reviewer #1: Yes

Reviewer #2: Yes

**********

3. Has the statistical analysis been performed appropriately and rigorously?

Reviewer #1: Yes

Reviewer #2: Yes

**********

4. Have the authors made all data underlying the findings in their manuscript fully available?

The PLOS Data policy requires authors to make all data underlying the findings described in their manuscript fully available without restriction, with rare exception (please refer to the Data Availability Statement in the manuscript PDF file). The data should be provided as part of the manuscript or its supporting information, or deposited to a public repository. For example, in addition to summary statistics, the data points behind means, medians and variance measures should be available. If there are restrictions on publicly sharing data—e.g. participant privacy or use of data from a third party—those must be specified.

Reviewer #1: Yes

Reviewer #2: Yes

**********

5. Is the manuscript presented in an intelligible fashion and written in standard English?

PLOS ONE does not copyedit accepted manuscripts, so the language in submitted articles must be clear, correct, and unambiguous. Any typographical or grammatical errors should be corrected at revision, so please note any specific errors here.

Reviewer #1: Yes

Reviewer #2: Yes

**********

6. Review Comments to the Author

Please use the space provided to explain your answers to the questions above. You may also include additional comments for the author, including concerns about dual publication, research ethics, or publication ethics. (Please upload your review as an attachment if it exceeds 20,000 characters)

Reviewer #1: Dear Authors. What were the results of balloon angioplasties of iatrogenic CoA and LPA stenosis? Was it effecive?

Reviewer #2: All the comments have been addressed, can be accepted to publish. Overall article is good and has addressed the shortcomings.

**********

7. PLOS authors have the option to publish the peer review history of their article (what does this mean?). If published, this will include your full peer review and any attached files.

If you choose “no”, your identity will remain anonymous but your review may still be made public.

**Do you want your identity to be public for this peer review?**  For information about this choice, including consent withdrawal, please see our Privacy Policy.

Reviewer #1: **Yes:**  Michał Gałeczka

Reviewer #2: No

**********

[NOTE: If reviewer comments were submitted as an attachment file, they will be attached to this email and accessible via the submission site. Please log into your account, locate the manuscript record, and check for the action link "View Attachments". If this link does not appear, there are no attachment files.]

To ensure your figures meet our technical requirements, please review our figure guidelines: https://journals.plos.org/plosone/s/figures

You may also use PLOS’s free figure tool, NAAS, to help you prepare publication quality figures: https://journals.plos.org/plosone/s/figures#loc-tools-for-figure-preparation.

NAAS will assess whether your figures meet our technical requirements by comparing each figure against our figure specifications.

# Author comment: Author response to Decision Letter 2

**DOI:** 10.1371/journal.pone.0354122.r004

22 May 2026

Manuscript ID: PONE-D-26-07135

Title: Factors associated with procedural difficulty and major adverse events during transcatheter closure of the patent ductus arteriosus

Response to the Editor and Reviewers

Reviewer comment:

“What were the results of balloon angioplasties of iatrogenic CoA and LPA stenosis? Was it effective?”

Response:

Thank you for this valuable comment. Among the five patients with left pulmonary artery (LPA) stenosis, spontaneous improvement was observed in three patients during follow-up and no intervention was required. The remaining two patients had peak pressure gradients of approximately 40 mmHg and underwent balloon angioplasty, resulting in hemodynamic improvement with residual gradients of approximately 30 mmHg. These patients remain under follow-up, and further intervention such as LPA stenting may be considered if progressive stenosis or right ventricular hypertension develops.

Regarding iatrogenic coarctation of the aorta, both patients had mild mean pressure gradients of approximately 10–20 mmHg and are currently being monitored clinically for systemic hypertension and progression of the lesion without additional intervention.

As these events were limited in number and detailed long-term follow-up outcomes were beyond the predefined objectives of the present study, we have not incorporated these additional data into the revised manuscript.

Attachment Submitted filename: Response to Reviewers 2nd.docx

# Peer review: Decision Letter 2

**DOI:** 10.1371/journal.pone.0354122.r005

**Authors:** Jennifer Tucker, Eyüp Serhat Çalık, Eyüp Serhat Çalık

6 Jul 2026

Factors associated with procedural difficulty and major adverse events during transcatheter closure of the patent ductus arteriosus

PONE-D-26-07135R2

Dear Dr. Jarutach,

We’re pleased to inform you that your manuscript has been judged scientifically suitable for publication and will be formally accepted for publication once it meets all outstanding technical requirements.

Within one week, you’ll receive an e-mail detailing the required amendments. When these have been addressed, you’ll receive a formal acceptance letter and your manuscript will be scheduled for publication.

An invoice will be generated when your article is formally accepted. Please note, if your institution has a publishing partnership with PLOS and your article meets the relevant criteria, all or part of your publication costs will be covered. Please make sure your user information is up-to-date by logging into Editorial Manager at Editorial Manager® and clicking the ‘Update My Information' link at the top of the page. For questions related to billing, please contact billing support.

If your institution or institutions have a press office, please notify them about your upcoming paper to help maximize its impact. If they’ll be preparing press materials, please inform our press team as soon as possible -- no later than 48 hours after receiving the formal acceptance. Your manuscript will remain under strict press embargo until 2 pm Eastern Time on the date of publication. For more information, please contact onepress@plos.org.

Kind regards,

Eyüp Serhat Çalık

Academic Editor

PLOS One

Additional Editor Comments (optional):

Reviewers' comments:

Reviewer's Responses to Questions

**Comments to the Author**

1. If the authors have adequately addressed your comments raised in a previous round of review and you feel that this manuscript is now acceptable for publication, you may indicate that here to bypass the “Comments to the Author” section, enter your conflict of interest statement in the “Confidential to Editor” section, and submit your "Accept" recommendation.

Reviewer #1: All comments have been addressed

**********

2. Is the manuscript technically sound, and do the data support the conclusions?

The manuscript must describe a technically sound piece of scientific research with data that supports the conclusions. Experiments must have been conducted rigorously, with appropriate controls, replication, and sample sizes. The conclusions must be drawn appropriately based on the data presented.

Reviewer #1: Yes

**********

3. Has the statistical analysis been performed appropriately and rigorously?

Reviewer #1: Yes

**********

4. Have the authors made all data underlying the findings in their manuscript fully available?

The PLOS Data policy requires authors to make all data underlying the findings described in their manuscript fully available without restriction, with rare exception (please refer to the Data Availability Statement in the manuscript PDF file). The data should be provided as part of the manuscript or its supporting information, or deposited to a public repository. For example, in addition to summary statistics, the data points behind means, medians and variance measures should be available. If there are restrictions on publicly sharing data—e.g. participant privacy or use of data from a third party—those must be specified.

Reviewer #1: Yes

**********

5. Is the manuscript presented in an intelligible fashion and written in standard English?

PLOS ONE does not copyedit accepted manuscripts, so the language in submitted articles must be clear, correct, and unambiguous. Any typographical or grammatical errors should be corrected at revision, so please note any specific errors here.

Reviewer #1: Yes

**********

6. Review Comments to the Author

Please use the space provided to explain your answers to the questions above. You may also include additional comments for the author, including concerns about dual publication, research ethics, or publication ethics. (Please upload your review as an attachment if it exceeds 20,000 characters)

Reviewer #1: I encourage the Authors to incoroporate this resuts (protrusion observation/angioplasty treatment, etc.) into discussion, as those findings are especially useful for clinicians.

**********

7. PLOS authors have the option to publish the peer review history of their article (what does this mean?). If published, this will include your full peer review and any attached files.

If you choose “no”, your identity will remain anonymous but your review may still be made public.

**Do you want your identity to be public for this peer review?**  For information about this choice, including consent withdrawal, please see our Privacy Policy.

Reviewer #1: No

**********

# Editor report: Acceptance letter

**DOI:** 10.1371/journal.pone.0354122.r006

**Authors:** Jennifer Tucker, Eyüp Serhat Çalık, Eyüp Serhat Çalık

PONE-D-26-07135R2

PLOS One

Dear Dr. Jarutach,

I'm pleased to inform you that your manuscript has been deemed suitable for publication in PLOS One. Congratulations! Your manuscript is now being handed over to our production team.

At this stage, our production department will prepare your paper for publication. This includes ensuring the following:

* All references, tables, and figures are properly cited

* All relevant supporting information is included in the manuscript submission,

* There are no issues that prevent the paper from being properly typeset

You will receive further instructions from the production team, including instructions on how to review your proof when it is ready. Please keep in mind that we are working through a large volume of accepted articles, so please give us a few days to review your paper and let you know the next and final steps.

Lastly, if your institution or institutions have a press office, please let them know about your upcoming paper now to help maximize its impact. If they'll be preparing press materials, please inform our press team within the next 48 hours. Your manuscript will remain under strict press embargo until 2 pm Eastern Time on the date of publication. For more information, please contact onepress@plos.org.

You will receive an invoice from PLOS for your publication fee after your manuscript has reached the completed accept phase. If you receive an email requesting payment before acceptance or for any other service, this may be a phishing scheme. Learn how to identify phishing emails and protect your accounts at https://explore.plos.org/phishing.

If we can help with anything else, please email us at customercare@plos.org.

Thank you for submitting your work to PLOS ONE and supporting open access.

Kind regards,

PLOS ONE Editorial Office Staff

on behalf of

Dr. Eyüp Serhat Çalık

Academic Editor

PLOS One
